# Impact of polypharmacy on oral health in the elderly: challenges and management

**DOI:** 10.3389/fdmed.2026.1758771

**Published:** 2026-02-23

**Authors:** Sowmya Halasabalu Kalgeri, SunilKumar Bheemasamudra Balaraj, Ashwini Tumkur Shivakumar, Vidya G. Doddawad, Deepa Basapur Vijayakumar, Shalini H. S., Anupama Aradya, Parinitha Mysore Shankar, Nagabhushana Doggalli, Sumukh R. Bharadwaj

**Affiliations:** 1 Department of Conservative Dentistry and Endodontics, JSS Dental College and Hospital, JSS Academy of Higher Education and Research, Mysore, Karnataka, India; 2 Department of Surgical Gastroenterologist, JSS Medical College and Hospital, JSS Academy of Higher Education and Research, Mysore, Karnataka, India; 3 Department of Oral Pathology and Microbiology, JSS Dental College and Hospital, JSS Academy of Higher Education and Research, Mysore, Karnataka, India; 4 Department of Oral and Maxillofacial Surgery, JSS Dental College and Hospital, JSS Academy of Higher Education and Research, Mysore, Karnataka, India; 5 Department of Periodontics, Subbaiah Institute of Dental Sciences, Shivamogga, Karnataka, India; 6 Department of Prosthodontics and Crown & Bridge, JSS Dental College and Hospital, JSS Academy of Higher Education and Research, Mysore, Karnataka, India; 7 Department of Oral Medicine and Radiology, JSS Dental College and Hospital, JSS Academy of Higher Education and Research, Mysore, Karnataka, India; 8 Private Practitioner, Mysore, Karnataka, India

**Keywords:** geriatric dentistry, interprofessional care, medication-related complications, oral health, polypharmacy, xerostomia

## Abstract

With increasing life expectancy and multimorbidity, polypharmacy—commonly defined as the concurrent use of five or more medications—has become highly prevalent in older adults and poses substantial risks for oral health. This narrative review aimed to synthesize contemporary evidence on the epidemiology, pharmacological mechanisms, and oral manifestations of polypharmacy in geriatric populations, and to propose an interprofessional management framework for dental practitioners in 2025 care settings. Recent epidemiological data indicate that polypharmacy affects more than 30%–44% of older adults globally, with even higher rates among those with cardiovascular disease, diabetes, and multimorbidity. Medications with anticholinergic burden, as well as many antihypertensive and psychotropic agents, are strongly associated with salivary gland hypofunction and xerostomia, which in turn contribute to rampant caries, oral candidiasis, mucosal lesions, dysgeusia, periodontal complications, and drug-induced gingival overgrowth. Diagnostic and therapeutic challenges are amplified by underreporting of symptoms, cognitive impairment, and fragmented medical–dental care pathways. Evidence supports a preventive and interdisciplinary approach that includes structured medication review and deprescribing in collaboration with physicians and pharmacists, optimization of salivary function using sialogogues and saliva substitutes, prescription of high-fluoride toothpastes, and tailored oral hygiene and dietary counseling with caregiver engagement. Dentists are strategically positioned to detect medication-related oral conditions, trigger timely medication optimization, and coordinate ongoing care. A structured, multidisciplinary model integrating dental, medical, and pharmacy services is essential to mitigate the oral and systemic consequences of polypharmacy and to preserve function and quality of life in aging populations.

## Introduction

Polypharmacy, commonly described as the simultaneous use of five or more medications, and hyperpolypharmacy, referring to an even higher count of concurrent medications, are increasingly prevalent in aging populations managing multiple chronic conditions (1). The aging population has witnessed a significant increase in the prevalence of polypharmacy due to the management of chronic systemic conditions (2). While polypharmacy aims to improve systemic health, its potential adverse effects on oral health are often overlooked. Medications such as anticholinergics, antidepressants, and antihypertensives have been linked to detrimental impacts on oral health (3). Storbeck et al. reported that 38.5% of older adults (mean age 74 years) in a special care dentistry cohort experienced xerostomia, with a clear dose-dependent relationship with the number of medications used (4). With these medications commonly implicated in altered taste, and increased susceptibility to dental caries and periodontal diseases. These oral health challenges not only compromise the quality of life but also pose difficulties in maintaining oral hygiene in elderly individuals. Medication-induced salivary hypofunction disrupts the oral microbiome, while sensory alterations like dysgeusia further compromise nutritional intake and hygiene practices (5, 6).

Emphasis is placed on actionable insights for dental practitioners, highlighting their integral role in identifying and addressing medication-related oral complications in the elderly. Dentists are frequently the first to observe medication-related changes, yet these manifestations may be dismissed or misattributed to aging alone. This article examines the mechanistic pathways linking polypharmacy to oral health deterioration, identifies clinical management challenges, and proposes integrated care strategies for 2025 geriatric dental practice.

## Clinical relevance

Dentists must recognize polypharmacy as a critical risk multiplier for oral diseases in the elderly, necessitating interdisciplinary collaboration to mitigate complications.

## Objective

The reader will understand evidence-based approaches to manage polypharmacy-related oral health decline, including deprescribing protocols, preventive interventions, and coordinated care models.

## Methodology

This article is a narrative, evidence-based review of the literature addressing the impact of polypharmacy on oral health in older adults, with particular emphasis on xerostomia and its clinical management. A targeted literature search was conducted using PubMed/MEDLINE, Scopus, and Web of Science to identify relevant peer-reviewed publications published between January 2013 and March 2024. Key search concepts included polypharmacy, geriatric oral health, xerostomia, salivary gland hypofunction, medication-induced oral effects, and multidisciplinary care. Only articles published in English were considered. In addition, the reference lists of selected articles and relevant reviews were manually screened to identify additional pertinent studies. The included evidence was synthesized descriptively to provide an up-to-date overview of current knowledge, clinical implications, and gaps in the literature.

## Epidemiology and burden of polypharmacy

The prevalence of polypharmacy has been increasing steadily over the past two decades. In the United States, for example, the prevalence among adults rose from 8.2% in 1999–2000 to 17.1% in 2017–2018 (7). Among adults aged 65 and older, the prevalence is much higher, reaching 44.1% in recent years (7, 8). Globally, similar trends are seen, driven by aging populations and the rising burden of chronic diseases that require long-term medication management (1, 8).

Polypharmacy is especially common in individuals with heart disease, diabetes, and other chronic conditions, with prevalence rates in these groups exceeding 50% in some studies (7). Hospitalized patients and those with multimorbidity are at even higher risk, and the prevalence of “excessive polypharmacy” (use of ≥10 drugs) can exceed 10% in older populations (8, 9).

Clinical Consequences of polypharmacy are strongly associated with increased risks of adverse drug events, medication errors, drug-drug interactions, and potentially inappropriate prescribing (7, 9, 10). The odds of a medication error rise by 16% for each additional drug prescribed (10). Serious drug-drug interactions occur in about 13% of patients with polypharmacy, and more than two-thirds of those on 10 or more medications have at least one potentially serious interaction (10). Despite the oral cavity's sensitivity to systemic drugs, oral complications remain underreported and underprioritized in broader geriatric care strategies. Table 1 provides the common drugs in polypharmacy and their oral manifestation.

**Table 1:** Common drug classes in polypharmacy and their oral manifestations.

Drug class	Common drugs in elderly	Oral adverse effects (elderly)	Polypharmacy-amplified mechanism in ageing	Clinical dental implications
Anticholinergics	Oxybutynin, Trihexyphenidyl	Severe xerostomia, burning mouth, denture intolerance	Age-related ↓ baseline salivary gland reserve + cumulative M3 receptor blockade from multiple drugs → profound hyposalivation	Rampant caries, denture stomatitis, difficulty in swallowing and speech
ACE Inhibitors	Enalapril, Lisinopril	Xerostomia, lichenoid reactions, dysgeusia	↑ Bradykinin accumulation + immunosenescence → exaggerated mucosal inflammatory response	Painful erosive lesions, poor oral intake
β-Blockers	Metoprolol, Atenolol	Dry mouth, taste alteration	Reduced autonomic adaptability in elderly → amplified β-receptor blockade on salivary glands	Reduced saliva buffering → caries risk
Calcium Channel Blockers	Amlodipine, Nifedipine	Gingival overgrowth	Drug accumulation due to ↓ hepatic clearance + synergism with cyclosporine → ↑ fibroblast activity and ECM deposition	Pseudo-pockets, plaque retention, periodontal breakdown
Tricyclic Antidepressants	Amitriptyline	Marked xerostomia, mucosal soreness	Strong antimuscarinic effect combined with age-related neuronal decline → severe salivary suppression	Burning mouth syndrome, candidiasis
SSRIs	Sertraline, Fluoxetine	Mild–moderate xerostomia, taste disturbance	Altered central serotonergic pathways + polypharmacy interaction affecting salivary reflexes	Appetite loss, nutritional compromise
Antipsychotics	Haloperidol, Risperidone	Drooling or dryness, candidiasis	Dopaminergic blockade + reduced oral motor control → saliva pooling or dysfunction	Aspiration risk, angular cheilitis
Antiepileptics	Phenytoin	Gingival hyperplasia	Long-term use + impaired collagen turnover in ageing tissues → exaggerated gingival response	Compromised oral hygiene, periodontal disease
Immunosuppressants	Cyclosporine	Gingival enlargement, opportunistic infections	Calcineurin inhibition + immunosenescence → ↑ fibroblast stimulation and ↓ host defense	Recurrent infections, delayed healing
NSAIDs	Ibuprofen, Diclofenac	Oral ulcers, mucositis	↓ Prostaglandin synthesis + reduced mucosal repair capacity in elderly	Painful lesions, difficulty wearing prostheses
Bisphosphonates	Alendronate, Zoledronic acid	MRONJ	Profound suppression of bone remodeling + ↓ angiogenesis + microtrauma accumulation in ageing jawbone	High risk after extractions or implant placement

Polypharmacy is linked to higher rates of hospitalizations, rehospitalizations, disability, cognitive decline, and mortality (7, 9, 11).

## Understanding polypharmacy in the elderly

Polypharmacy in the elderly is a growing clinical concern driven by multimorbidity and age-related physiological changes that significantly alter both pharmacokinetics and pharmacodynamics. Ageing is associated with reduced renal clearance, diminished hepatic metabolism, altered plasma protein binding, and changes in body composition, all of which increase drug bioavailability and prolong drug half-life, thereby heightening susceptibility to adverse drug reactions and drug–drug interactions. In parallel, pharmacodynamic changes—including altered receptor sensitivity and impaired homeostatic mechanisms—render older adults more vulnerable to exaggerated or atypical drug responses even at standard therapeutic doses. These factors contribute substantially to the high prevalence of adverse drug events observed in geriatric populations (12).

An additional and often under-recognized contributor to polypharmacy is the phenomenon of prescribing cascades, wherein medication-induced side effects are misinterpreted as new disease entities, leading to the initiation of further medications and escalation of drug burden. Such cascades are particularly problematic in older adults, as nonspecific symptoms such as xerostomia, dizziness, fatigue, or cognitive changes are frequently attributed to ageing or comorbid disease rather than medication effects (13).

For dental professionals, this complexity poses diagnostic challenges, as oral manifestations—including salivary gland dysfunction, mucosal alterations, increased caries risk, delayed wound healing, and heightened susceptibility to infections—may arise from polypharmacy rather than primary oral or systemic pathology. Distinguishing medication-related oral changes from age-related degeneration or systemic disease is therefore critical for appropriate diagnosis, treatment planning, and referral. Recent literature emphasizes that comprehensive medication review, interprofessional collaboration, and awareness of prescribing cascades are essential to mitigate polypharmacy-related oral health deterioration and improve overall care outcomes in the ageing population (5, 13).

## Mechanisms linking polypharmacy to oral health deterioration

Polypharmacy in older adults is closely linked to oral health deterioration through several intertwined biological and pharmacological mechanisms. Many commonly prescribed medications—such as anticholinergics, antidepressants, diuretics, and antihypertensives—reduce salivary gland output, leading to xerostomia (dry mouth). This reduction in saliva not only impairs the natural cleansing of the oral cavity but also increases the risk of dental caries, oral candidiasis, and mucosal fragility (6, 14).

Drug-induced hyposalivation occurs through distinct pharmacological pathways depending on the drug class. Anticholinergic agents and tricyclic antidepressants reduce salivary secretion via competitive antagonism of muscarinic M3 receptors on salivary acinar cells, leading to impaired intracellular calcium signaling necessary for fluid secretion. Selective serotonin reuptake inhibitors alter central and peripheral serotonergic pathways that regulate salivary reflexes, resulting in functional salivary hypofunction (15–17).

β-adrenergic blockers reduce salivary output through both hemodynamic and cellular mechanisms. Blockade of β1-adrenoceptors decreases cardiac output and salivary gland perfusion, while inhibition of β-adrenergic signaling prevents upregulation of the Na^+^-K^+^-2Cl^−^ cotransporter in acinar cells, impairing chloride-dependent water movement into saliva. Thiazide diuretics reduce salivary secretion by inhibiting carbonic anhydrase in salivary acinar cells, leading to decreased bicarbonate availability and impaired salivary buffering. In older adults, these effects are cumulative and often synergistic due to the concurrent use of multiple xerogenic medications (18–20).

Additionally, cytotoxic drugs and corticosteroids can suppress local immune responses, resulting in oral mucosal ulcerations, increased susceptibility to infections, and delayed wound healing. The use of broad-spectrum antibiotics disrupts the balance of oral microbiota, fostering the overgrowth of opportunistic pathogens and further contributing to oral infections (6, 21).

Furthermore, medications such as benzodiazepines and antipsychotics may impair neuromuscular coordination, making it more difficult for patients to maintain adequate oral hygiene and increasing the risk of periodontal disease (22). Drugs that induce gastroesophageal reflux or vomiting, such as certain antihypertensives and chemotherapeutic agents, can cause enamel erosion due to repeated acid exposure. Lastly, medications that alter taste, smell, or swallowing function—such as some antihypertensives and psychotropics—can negatively impact nutritional intake and mucosal health, compounding the risk of oral disease in polypharmacy (23). These mechanisms highlight the importance of regular oral health assessments and interdisciplinary care for elderly individuals on multiple medications.

## Specific oral health complications

Polypharmacy in geriatric patients is closely linked to a spectrum of oral health issues, primarily due to the pharmacological effects of medications on salivary flow, mucosal integrity, and immune responses. The following are commonly observed complications:

Dry Mouth (Xerostomia): Reported in up to 30% of elderly individuals on multiple medications, xerostomia is a frequent adverse effect associated with antihypertensives, antidepressants, anticholinergics, and diuretics. It significantly increases the risk of rampant dental caries, halitosis, mucosal irritation, and oral discomfort, compromising both oral and general well-being (6, 24).
Oral Candidiasis: This opportunistic fungal infection is particularly prevalent in denture-wearing elderly patients, especially those receiving inhaled corticosteroids or broad-spectrum antibiotics. Reduced salivary flow and altered oral flora further exacerbate susceptibility (3, 25).
Drug-Induced Gingival Enlargement: Commonly associated with medications such as calcium channel blockers (e.g., nifedipine), phenytoin, and cyclosporine, gingival overgrowth complicates oral hygiene maintenance and may necessitate surgical intervention (3, 25).
Glossodynia and Dysgeusia: Burning mouth syndrome and altered taste perception are frequent complaints in the elderly on polypharmacy, particularly those using selective serotonin reuptake inhibitors (SSRIs), angiotensin-converting enzyme (ACE) inhibitors, and chemotherapeutic agents. These symptoms adversely affect nutrition and quality of life (3, 26).
Mucositis and Oral Ulceration: These painful lesions are often drug-induced, notably by NSAIDs, chemotherapeutic agents, and bisphosphonates. They can lead to secondary infections and compromise the patient's ability to eat and speak comfortably (3).
Denture-Related Lesions: Polypharmacy-induced mucosal dryness, fragility, and impaired healing contribute to a higher incidence of denture stomatitis, traumatic ulcers, and epulis fissuratum. Poor denture hygiene and prolonged wearing time further aggravate these lesions (3).

Early recognition and management of these oral manifestations are crucial for maintaining both oral health and overall systemic health in elderly patients who are receiving multiple medications. Table 2 summarizes Oral Health Complications Associated with Polypharmacy in the Elderly.

**Table 2:** Oral health complications associated with polypharmacy in the elderly.

Oral condition	Clinical features	Associated drug classes	Contributing factors
Xerostomia (Dry mouth)	Dryness, sticky sensation, difficulty swallowing, halitosis, caries risk	Anticholinergics, antidepressants, antihypertensives, diuretics	Reduced salivary flow, age-related salivary gland hypofunction
Oral candidiasis	White patches, burning sensation, erythema, angular cheilitis	Inhaled corticosteroids, broad-spectrum antibiotics	Denture use, immunosuppression, dry mucosa
Gingival enlargement	Gingival overgrowth, bleeding, interference with oral hygiene	Calcium channel blockers (e.g., nifedipine), phenytoin, cyclosporine	Inadequate plaque control, long-term use
Glossodynia & dysgeusia	Burning sensation, altered or metallic taste	Antidepressants (SSRIs), ACE inhibitors, chemotherapeutics	Neuropathy, nutritional deficiencies, mucosal sensitivity
Mucositis and ulcers	Erythematous, painful mucosa; ulcerations; secondary infections	Chemotherapeutics, NSAIDs, bisphosphonates	Mucosal toxicity, immune suppression
Denture-related lesions	Traumatic ulcers, stomatitis, epulis fissuratum	Indirectly from medications causing mucosal dryness or fragility	Poor denture hygiene, continuous wear, xerostomia

## Challenges in diagnosis and management

Managing oral complications arising from polypharmacy in older adults is fraught with significant challenges. Symptom underreporting is common, as many elderly patients perceive dry mouth, altered taste, or oral discomfort as inevitable aspects of aging and therefore do not mention them during medical consultations (27). Diagnostic ambiguity further complicates care, since oral manifestations such as mucosal lesions, ulcers, or candidiasis can easily be mistaken for other systemic or local conditions, leading to misdiagnosis or delayed intervention (28). Recent reviews on polypharmacy and oral health in elders echo these concerns, emphasizing that cross-sectional designs relying on self-report likely underestimate true disease burden and that standardized xerostomia/oral examination protocols are rarely implemented in routine geriatric assessments (29–31).

Cognitive impairment—prevalent in conditions like dementia—poses another barrier, making it difficult to obtain accurate histories and diminishing patients' ability to maintain oral hygiene or recognize changes in their oral health (32). The lack of integration between medical and dental care remains a persistent issue, with poor communication and fragmented records impeding coordinated management of polypharmacy and its oral sequelae (3). Finally, limited dental access due to mobility challenges, transportation difficulties, and financial constraints further reduces the likelihood that older adults will seek or receive timely dental care, exacerbating the risk of undiagnosed and untreated oral disease (33).

Together, these findings from recent literature reinforce that the challenges described in this review are not isolated observations but reflect systemic patterns; they underscore the need for integrated medical–dental care models, routine oral screening within geriatric medicine, and targeted policies to reduce financial and logistical barriers for older adults on polypharmacy (34, 35).

## Compounding challenges

Beyond the direct effects of polypharmacy and oral health management, several additional factors further complicate the oral health landscape in older adults (Table 3). Functional decline—such as reduced manual dexterity—impairs the ability to perform effective plaque control and maintain dental prostheses, increasing the risk of oral disease (28). Recent studies confirm that polypharmacy accelerates functional decline in activities of daily living, including oral self-care, with odds ratios of 1.5–2.0 for dependency in elders taking ≥5 medications (36, 37).

**Table 3:** Compounding challenges in polypharmacy and oral health in the elderly.

Challenge	Description	Impact on oral health
Functional decline	Reduced hand dexterity due to arthritis, stroke, or neuromuscular disorders	Impaired brushing/flossing, poor denture maintenance
Nutritional deficiencies	Difficulty eating due to oral discomfort, poor appetite, or missing teeth	Delayed healing, mucosal atrophy, increased susceptibility to infections
Social isolation	Living alone, lack of social support, or depression	Neglect of oral hygiene routines and missed dental appointments
Language & health literacy barriers	Inability to comprehend medication instructions or dental care guidelines	Misuse of medications, inadequate hygiene practices, delayed care seeking

Nutritional deficiencies often arise as poor oral function and decreased appetite lead to inadequate dietary intake, which in turn negatively affects the health of oral tissues and impairs healing (24). A 2025 cross-sectional analysis of long-term care residents demonstrated that polypharmacy (≥5 drugs) independently predicts malnutrition, with xerogenic medications compounding reduced food intake and micronutrient deficits (38).

Social isolation is another significant challenge; elderly individuals living alone are more likely to neglect daily oral hygiene routines and miss dental appointments, exacerbating oral health problems. Additionally, language and health literacy barriers can limit a patient's understanding of the effects of medications and the importance of oral care, hindering effective self-management and communication with healthcare providers (33). Recent qualitative and epidemiological reviews align with these observations, noting that low literacy correlates with suboptimal medication adherence and oral hygiene in multimorbid elders (*r* = −0.42 for literacy scores and plaque index), and calling for simplified educational tools and multilingual interventions (39). These overlapping challenges underscore the need for holistic, multidisciplinary approaches to the care of older adults with complex medication regimens.

## Strategies for oral healthcare providers

**Figure 1:**
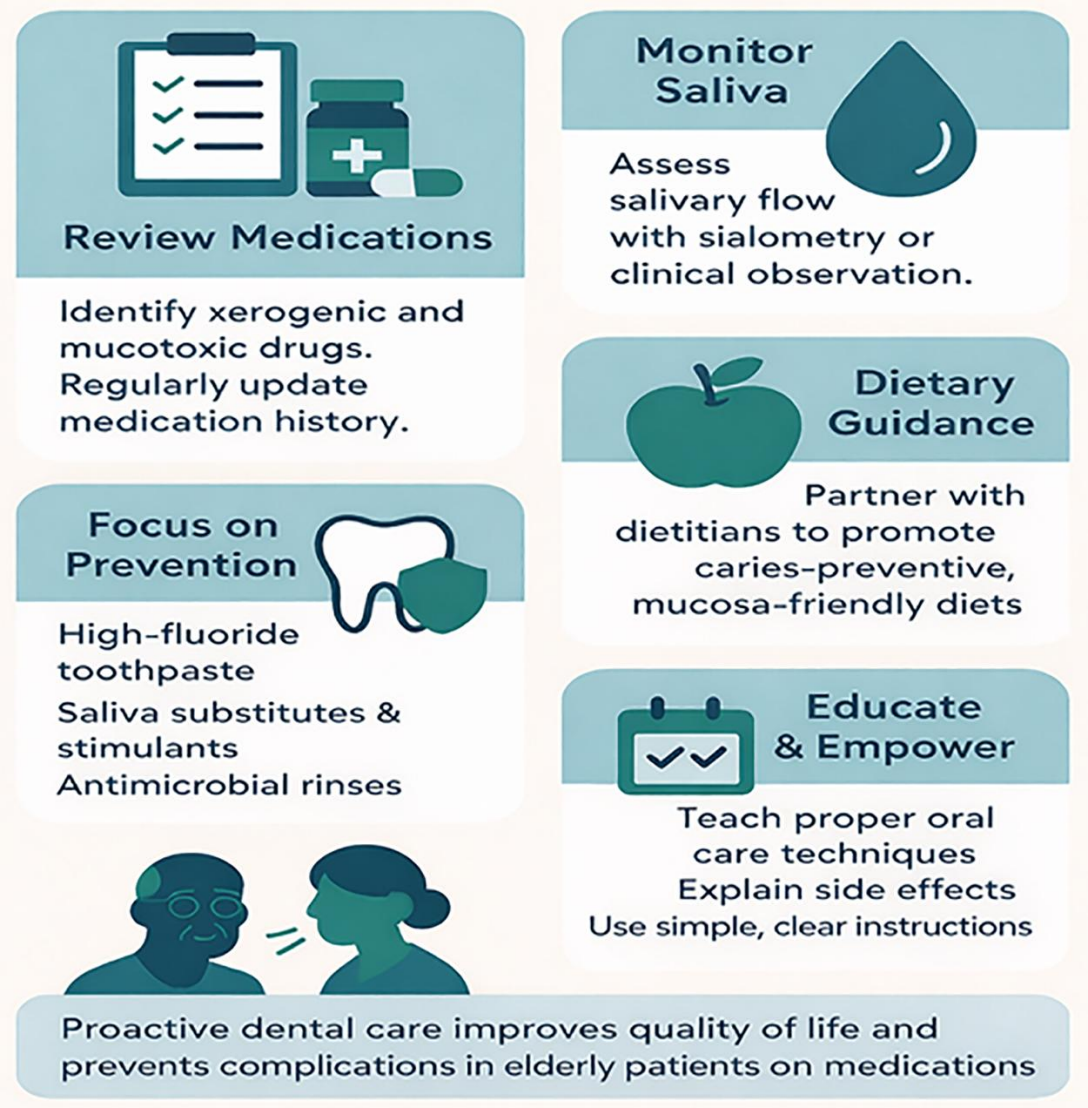
Managing oral health in older adults on polypharmacy.

Dental professionals play a pivotal role in mitigating the oral health risks associated with polypharmacy in older adults by implementing a structured and proactive care approach, yet recent evidence indicates both strong support for and important refinements to this model (Figure 1). A thorough medication history and regular review are essential, with particular attention to identifying xerogenic and mucotoxic agents that may compromise oral health (24). Regular assessment of salivary flow—using sialometry or validated clinical scoring systems—enables early detection of hyposalivation and informs tailored management strategies (32). The emphasis on preventive care — including high-fluoride toothpaste, saliva substitutes, and antimicrobial rinses — aligns with systematic reviews reporting benefits of professional and self-applied fluoride in reducing coronal and root caries, though findings regarding certain antimicrobial agents such as chlorhexidine varnish are mixed and appear more effective in high-risk individuals than in unselected populations (29).

Dietary counseling should be integrated into care plans, ideally in collaboration with dietitians, to promote diets that are both caries-preventive and gentle on the oral mucosa (28). The frequency of recall visits should be individualized based on each patient's risk status and clinical findings, ensuring timely intervention and ongoing support. Finally, the critical role of caregiver and patient education is reinforced by evidence demonstrating improved oral hygiene outcomes when caregivers are engaged in daily care and trained in oral assessment protocols; however, the literature underscores a need for more robust interprofessional frameworks and outcome-focused evaluations of multidisciplinary care models, as most current research remains descriptive rather than interventional (40). Emerging concepts such as deprescribing initiatives and clinical decision support tools for medication management, which aim to reduce medication burden and associated xerostomic effects, are gaining traction in geriatric medicine and represent under-represented but promising areas for dental integration (41). Collectively, these findings support the core elements of the structured care approach while highlighting the need for standardized assessment methods, stronger evidence for some preventive strategies, and enhanced interdisciplinary collaboration to advance evidence-based practice in polypharmacy-related oral health care for older adults.

## Management strategies

Effective treatment of oral complications in elderly patients on polypharmacy should target both the underlying pharmacological causes and provide symptomatic relief. An individualized, evidence-based approach is essential.

Management of xerostomia in older adults with polypharmacy should fundamentally focus on identifying and minimizing xerogenic medications rather than introducing additional systemic drugs. Recent literature consistently emphasizes that drug-induced xerostomia is best addressed through comprehensive medication review, deprescribing where appropriate, or substitution with agents having a lower xerogenic potential, undertaken in collaboration with the prescribing physician or pharmacist (40, 41). In contrast to earlier symptom-driven approaches, contemporary reviews caution against the routine use of systemic sialogogues such as pilocarpine and cevimeline in polypharmacy-related xerostomia, as their therapeutic indications are largely restricted to post-irradiation salivary gland hypofunction and autoimmune conditions such as Sjögren's syndrome, where residual glandular function exists (42). The evidence supporting their effectiveness in medication-induced xerostomia is weak, and their use may paradoxically worsen polypharmacy, increase adverse effects, and compromise adherence in frail older adults (40–42). Recent scoping and narrative reviews therefore advocate a shift toward etiologic management, prioritizing medication optimization over pharmacological escalation (40, 41). Non-pharmacological strategies, including topical salivary stimulation with sugar-free chewing gums or lozenges, adequate hydration, saliva substitutes, and oral moisturizers, are supported by moderate-quality evidence for improving subjective symptoms and oral comfort, although they do not restore salivary gland function (42). These measures are consistently recommended as first-line interventions due to their favorable safety profile. Furthermore, recent geriatric oral health literature highlights that dentists play a critical role in interdisciplinary care by identifying xerogenic burden and facilitating communication with medical teams, rather than independently prescribing systemic agents (40, 41, 43). Overall, compared with earlier management models, current evidence supports a conservative, interprofessional, and medication-focused strategy for polypharmacy-associated xerostomia, reinforcing the need to avoid unnecessary addition of systemic sialogogues in this population.

In cases of confirmed oral candidiasis, treatment includes topical agents such as *nystatin suspension, miconazole* oral gel, or systemic antifungals like *fluconazole* for resistant or widespread infections (36). Meticulous denture hygiene is also crucial in managing denture-associated candidiasis.

Gingival overgrowth often warrants collaboration with the patient's physician to explore possible substitution of the causative drug (such as *calcium channel blockers or phenytoin*), and in advanced cases, surgical intervention like gingivectomy may be necessary (37). In mucosal lesions, the first step is identifying and removing contributing local irritants, such as ill-fitting dentures, sharp cusps, or abrasive oral hygiene aids. Topical corticosteroids (e.g., *triamcinolone acetonide 0.1%)* can be prescribed to manage inflammatory lesions such as lichenoid reactions or mucositis (36).

Prosthodontic care should focus on regular denture hygiene instruction and timely adjustments to relieve mucosal trauma, especially in patients with dry mouth or fragile oral tissues (36). A multidisciplinary, patient-centered approach—incorporating preventive strategies, medication review, and patient education—is essential to optimize oral health outcomes in this vulnerable population

## Interprofessional collaboration

The complexity of managing oral health in older adults with polypharmacy underscores the necessity of interprofessional collaboration. Interprofessional collaboration is essential for effectively managing oral health challenges. Dentists play a critical frontline role in identifying adverse drug reactions (ADRs) manifesting in the oral cavity, such as xerostomia, candidiasis, gingival overgrowth, and mucosal ulcers. Their responsibilities include not only diagnosing and documenting these oral ADRs but also communicating findings to the broader healthcare team. Prompt documentation of these conditions should trigger communication with the patient's general practitioner or prescribing physician (44).

Effective interprofessional communication enables timely modifications to medication regimens and early referral when specialized care is needed. Dentists should also contribute to shared clinical records and actively participate in interprofessional meetings to ensure oral health is integrated into the broader care plan (45).

Pharmacists bring essential expertise in reviewing complex medication profiles, identifying xerogenic or mucotoxic drugs, and recommending safer alternatives where appropriate. Their role in medication reconciliation is particularly vital during transitions of care, such as hospital discharge or nursing home admission. By evaluating the cumulative anticholinergic burden and drug-drug interactions, pharmacists can work collaboratively with prescribers and dental professionals to reduce the risk of oral complications. Pharmacists are also well-positioned to educate patients on the oral side effects of medications and support adherence to oral care protocols involving medicated rinses or saliva substitutes (46).

Primary care physicians and geriatricians are central to coordinating deprescribing strategies when polypharmacy poses more harm than benefit, particularly when oral health deteriorates or patient compliance declines. They are responsible for evaluating systemic risks, adjusting therapeutic plans, and referring to dental professionals when oral conditions persist. Meanwhile, nurses and caregivers in home or institutional settings provide the hands-on support necessary for daily oral hygiene and for monitoring changes in oral health. Integrated electronic health records and structured shared care plans enhance coordination, enabling real-time updates among all stakeholders and preventing fragmented care (46, 47). Collaborative, patient-centered approaches not only improve oral outcomes but also reduce hospitalizations and enhance overall quality of life in older adults.

## Special considerations

Geriatric oral healthcare requires tailored strategies that address the unique needs of older adults across various settings. In individuals with dementia, simplified daily routines, visual aids, and structured caregiver training are essential to reduce anxiety and ensure consistent oral hygiene (48). As cognitive decline progresses, caregivers play a central role in maintaining oral health, highlighting the importance of their involvement and education (49). In end-of-life care, the dental focus shifts from restorative interventions to palliative management, prioritizing patient comfort, hydration, and control of mucosal symptoms such as dry mouth, candidiasis, and ulcerations to preserve dignity and quality of life (49). For elderly individuals residing in institutional settings such as nursing homes, implementing formal oral health policies, providing regular dental screenings, and offering ongoing staff training can address the frequently unmet oral health needs in this population (50). Finally, culturally sensitive care is vital to promote trust and adherence; understanding patient values, beliefs, and preferences—including traditional practices or dietary customs—can guide personalized and respectful care delivery.

## Future direction

Looking ahead, several promising directions can enhance oral health outcomes among older adults, particularly those affected by polypharmacy. The development and widespread adoption of standardized assessment tools for identifying and managing medication-induced oral conditions are urgently needed to ensure consistent and accurate diagnosis across care settings (25). Integrating dedicated polypharmacy modules into geriatric dental curricula will better prepare dental professionals to recognize and address the complex oral health challenges associated with multiple medication use (1). Policy advocacy is also crucial, particularly for the inclusion of oral health assessments in primary care screening protocols, which would facilitate early detection and intervention for oral complications related to systemic medications (14). The integration of artificial intelligence (AI) holds great promise, as AI-driven tools can help predict oral health risks in patients with complex polypharmacy profiles, enabling more personalized and proactive care (51). Finally, there is a pressing need for longitudinal studies to clarify causal relationships between polypharmacy and oral health, as well as to evaluate the effectiveness of targeted interventions over time. By pursuing these directions, the field can move toward more comprehensive, evidence-based, and patient-centered oral health care for the aging population.

## Conclusion

Polypharmacy is a hallmark of geriatric healthcare, exerting a profound influence on oral health outcomes in older adults. Dental professionals, often among the first to detect medication-induced oral changes, are uniquely positioned to facilitate early recognition, prevention, and collaborative management of these conditions. Adopting an integrated, patient-centered approach is imperative to effectively reduce oral morbidity and improve overall quality of life in the elderly population.
